# Beneficial Effect of Melatonin Alone or in Combination with Glatiramer Acetate and Interferon β-1b on Experimental Autoimmune Encephalomyelitis

**DOI:** 10.3390/molecules27134217

**Published:** 2022-06-30

**Authors:** Genaro Gabriel Ortíz, Ana Laura Briones-Torres, Gloria Benitez-King, Luis Javier González-Ortíz, Claudia Verónica Palacios-Magaña, Fermín Paul Pacheco-Moisés

**Affiliations:** 1 Department of Philosophical and Methodological Disciplines, University Health Sciences Center, University of Guadalajara, Guadalajara 44340, Jalisco, Mexico; genarogabriel@yahoo.com; 2 Department of Chemistry, University Center of Exact Sciences and Engineering, University of Guadalajara, Guadalajara 44430, Jalisco, Mexico; luisj.gonzalezo@academicos.udg.mx (L.J.G.-O.); claudia.palacios@academicos.udg.mx (C.V.P.-M.); 3 National Institute of Psychiatry Ramón de la Fuente Muñíz, Mexico City 14370, Mexico; bekin54@hotmail.com

**Keywords:** oxidative stress, inflammatory cytokine, membrane fluidity, antioxidant

## Abstract

Experimental autoimmune encephalomyelitis (EAE) is a relevant animal model of multiple sclerosis (MS). Oxidative stress and chronic inflammation play a major role in the pathogenesis of MS and EAE. Melatonin, a neurohormone, has potent anti-inflammatory properties. The aim of our study was to assess the therapeutic properties of melatonin alone or in combination with interferon β-1b (IFNβ-1b) or glatiramer acetate (GA) on EAE. EAE was induced in male Sprague-Dawley rats with an intraperitoneal injection of a homogenate of spinal cord and pig brain. At day 10 post immunization, rats were euthanized, and their brains were immediately excised and processed to measure oxidative stress markers and membrane fluidity. In addition, proinflammatory cytokines were quantified in plasma. Melatonin alone or in combination with GA and IFNβ-1b inhibited the disease process of EAE and the synthesis of proinflammatory cytokines, caused a significant decrement in oxidative stress markers, and preserved the membrane fluidity in the motor cortex, midbrain, and spinal cord. The cumulative index score was significantly reduced in EAE rats treated with melatonin alone or in combination with GA and IFNβ-1b. In conclusion, our findings provide preclinical evidence for the use of melatonin as an adjuvant therapeutic treatment for MS.

## 1. Introduction

Multiple sclerosis (MS) is characterized by inflammation, demyelination, axonal loss, gliosis, and oxidative damage, which are associated with the overproduction of reactive free radicals. Experimental autoimmune encephalomyelitis (EAE) is an animal model of human MS and can be induced by inoculation with an encephalitogenic emulsion, consisting of nerve tissue or purified components of myelin and Freund’s complete adjuvant (CFA) [1]. EAE occurs with weight loss, progressive decrease in the tone of the tail, paraparesis, and incontinence, and it usually progresses to paraplegia and death of the animal, although, occasionally, a spontaneous recovery may occur. MS and EAE share many pathological and immune characteristics, such as autoreactivity of CD4^+^ Th1/lymphocytes directed against proteins present in myelinated membranes of the central nervous system (CNS) [2], the transference pattern of lymphocyte trafficking through the blood–brain barrier (BBB) into the CNS, lymphocyte infiltration, and axonal damage [3].

Oxidative stress plays a major role in the pathogenesis of MS and EAE, i.e., excess reactive oxygen species (ROS) and reactive nitrogen species (RNS), mainly generated by macrophages, which have been implicated as mediators of demyelination and axonal damage. In fact, neurons and oligodendrocytes are extremely susceptible to oxidative damage [4]. Thus, treatment with antioxidants might prevent propagation of tissue damage and improve both survival and neurological outcome [5].

Medical treatments approved for multiple sclerosis are usually immunomodulators, such as type I interferon beta (IFN-β) and glatiramer acetate (GA); in fact, no differences in clinical effect between the two treatments were shown [6], although the clinical impact of these data is currently being studied [7]. Interestingly, patients treated with IFN-β continue with inflammation and neurodegeneration [8]. Recently, it was described that mesenchymal stem cells transplanted in EAE mice induce neurogenesis and an anti-inflammatory response that ameliorates the symptoms of encephalomyelitis [9].

The effect of IFN-β is mediated through its membrane receptor (IFNAR), which activates the transcription of several genes, including interferon regulatory factors 7 and 9 (IRF7 and IRF9); those factors are also involved in the induction of endogenous type I IFN [10]. In addition, the effects of interferon β-1b (IFNβ-1b) include interference with T-cell activation, reduction in the production of proinflammatory cytokines, downregulation of antigen presentation, and inhibition of T-lymphocyte penetration into the CNS [11].

GA is a random copolymer of four amino acids present in myelin basic protein (glutamic acid, lysine, alanine, and tyrosine), which act as an immunomodulatory agent used in MS and EAE treatment [12], presenting some effects that are T-cell-independent [13]. Furthermore, GA diminishes microglial activation at specific brain areas [14], as well as TNF-α release by activated microglia [15]. GA increases neurotrophic factors such as brain-derived neurotrophic factor, involved in neuronal and glial cell survival, and it may mediate neuroprotection. In addition, GA induces remyelination and enhances neurogenesis [16].

Melatonin (*N*-acetyl-5-methoxytryptamine), a neurohormone that regulates daily cycles, in both animals and humans, is involved in multiple physiological actions such as energy metabolism, anti-inflammatory properties, and immunological activity. Melatonin is produced by the pineal gland, retina, gastrointestinal tract, macrophages, and other cell types [17]. Melatonin is also synthesized in plants and modulates several metabolic activities including carbon fixation, biosynthesis of plant hormones, and redox homeostasis [18]. High doses of exogenous melatonin have protective effects against biotic stress in plants [19], and they increase the synthesis of secondary metabolites with anti-oncogenic properties [20]. Interestingly, it has been reported that intake of melatonin from plant foods has beneficial effects in several animal models of degenerative diseases [18].

A potent anti-inflammatory property of melatonin is associated with its ability to act as a scavenger of exogenous and endogenous ROS and RNS. Furthermore, melatonin does not undergo auto-oxidation, redox cycling, or reactions producing hydroxyl radicals. In addition, melatonin does not have a toxic effect even when used in very high doses and for a long time, and it is well assimilated [21]. Exogenous melatonin has neuroprotective actions in several animal models [22,23]. These findings highlight the use of exogenous melatonin as an adjuvant therapy for degenerative diseases.

The effect of melatonin in an MS model was attributed to a decrease in peripheral and central Th1/Th17 responses and an increase in both Treg frequency and IL-10 synthesis. Thus, melatonin reduces the proinflammatory response [24]. In addition, melatonin protects the neuronal cytoskeletal integrity against the damage caused by free radicals and stimulates neurodevelopment in the adult brain [25,26]. In particular, this indolamine prompts neurogenesis and dendritogenesis to repair the circuitry loss caused by free radicals [27].

The aim of this study was to evaluate the physiological efficacy of melatonin, GA, and IFN β-1b either alone or in combination (melatonin - GA or melatonin - IFNβ-1b) on the clinical signs, proinflammatory cytokine levels, and oxidative stress markers in a rat model of MS.

## 2. Results

### 2.1. Clinical Score Assessment

**Figure 1:**
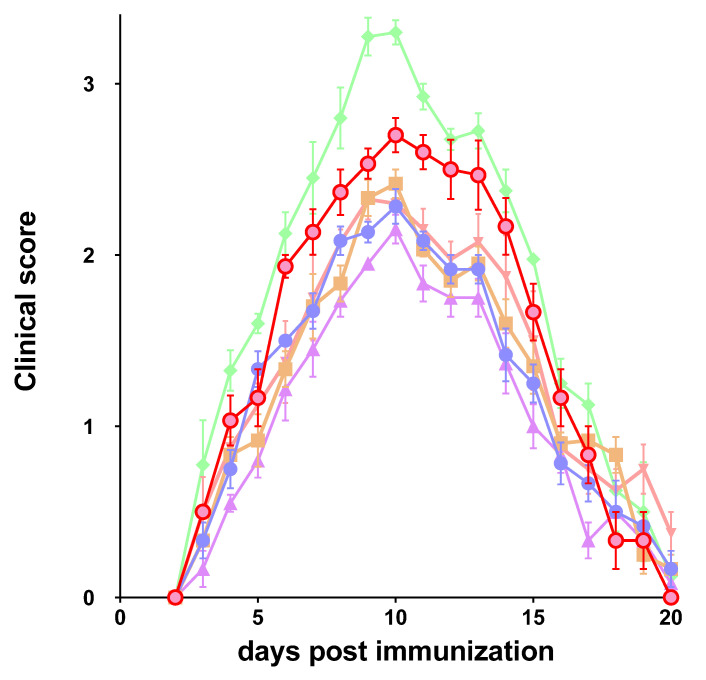
Clinical course of EAE in Sprague-Dawley rats. Rats showed the maximal signs on day 10. Rats were immunized and treated as indicated in Section 4. EAE immunized rats (♦); EAE immunized rats treated with glatiramer acetate (▼), interferon beta 1b (●), melatonin (▲), glatiramer acetate + melatonin (■), or interferon beta 1b + melatonin (●). Clinical scores are expressed as the mean ± SD.

**Figure 2:**
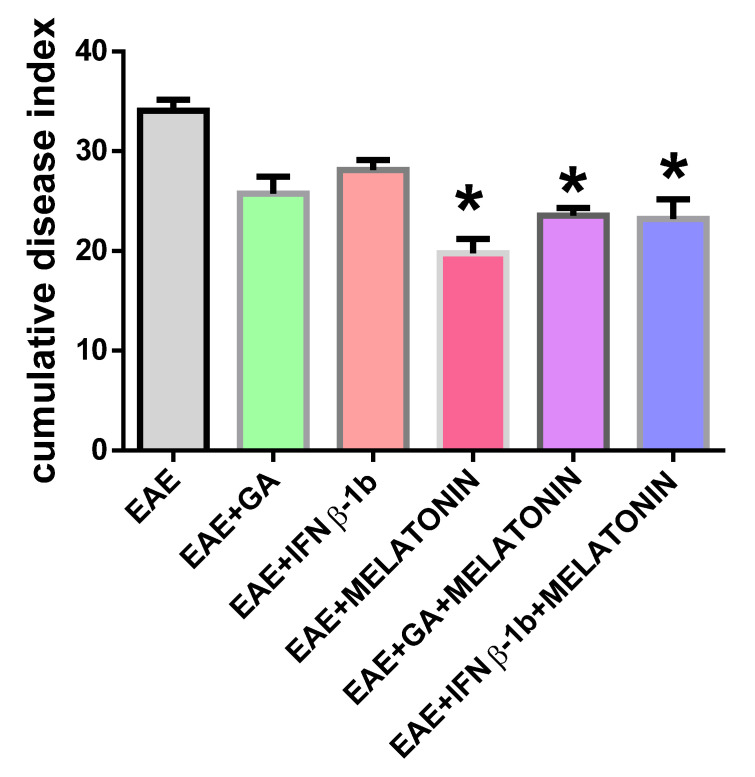
Cumulative disease index score. Scores are expressed as the mean ± SD. * indicates a significant difference from the EAE group (*p* < 0.05).

The degree of paralysis in EAE rats increased steadily during the first 9 days after the immunization. At day 20 post immunization, all rats were fully recovered. EAE rats treated with melatonin alone or in combination with GA and IFNβ-1b developed a lower disease progression rate, and the time of onset was significantly delayed compared with group 1 (control group with EAE). Treatments using GA and IFNβ-1b, alone or in combination with melatonin, also showed a decrease in clinical symptoms, although their effect was lower than that observed with the administration of melatonin alone (Figure 1). As shown in Figure 2, the cumulative index score was significantly reduced in EAE rats treated with melatonin alone or in combination with GA and IFNβ-1b.

### 2.2. Proinflammatory Cytokines

**Figure 3:**
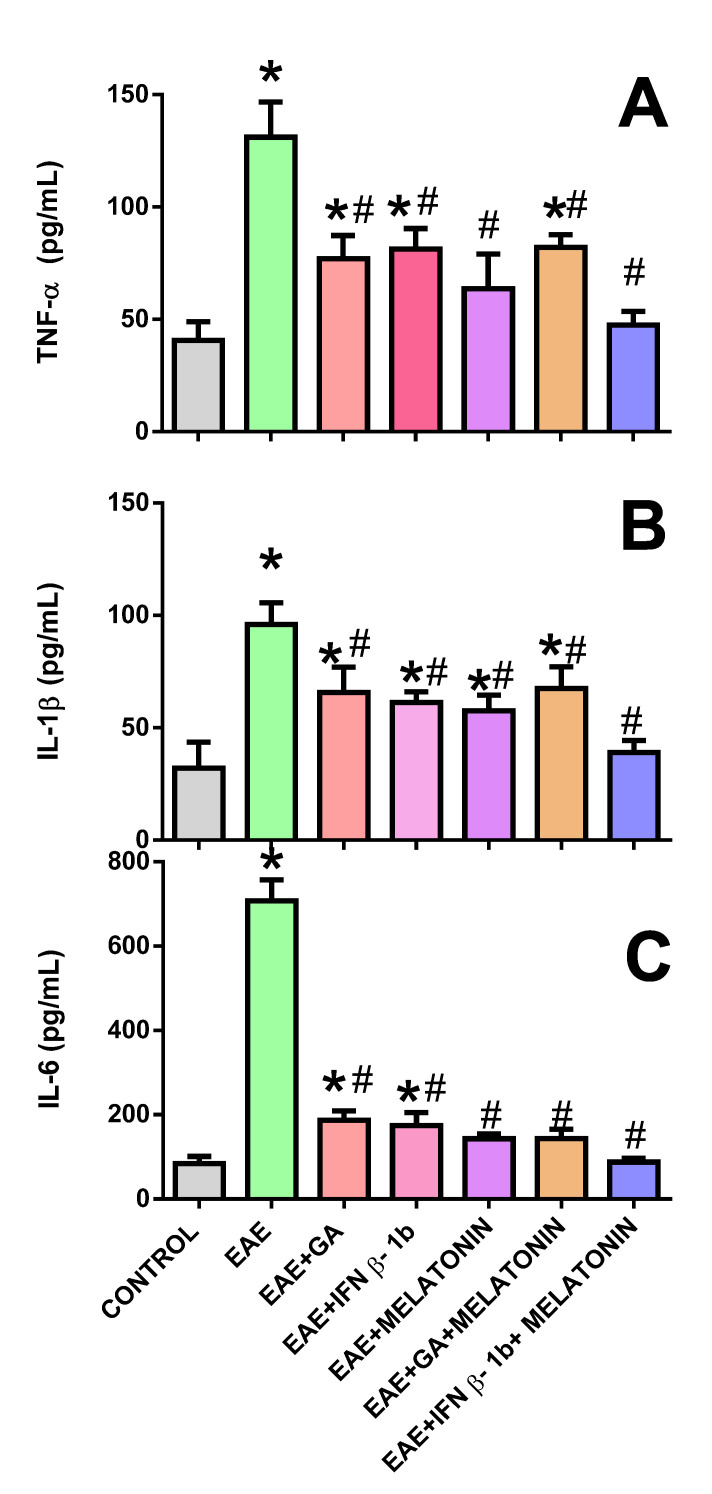
Plasma concentrations of TNFα (**A**), IL1β, (**B**), and IL6 (**C**) from rats immunized as indicated above and subjected to the indicated treatments. * indicates a significant difference from the control group (*p* < 0.05); # indicates a significant difference from the EAE group (*p* < 0.05).

In this study, plasma TNFα (Figure 3A) and IL-1β (Figure 3B) levels in EAE rats (group 1) were significantly higher than in the nonimmunized control group. In contrast, in the groups of rats with EAE that received treatment with GA, IFNβ-1b, melatonin, and GA + melatonin, the levels of TNFα and IL-1β were lower than in group 1, but these levels usually remained higher than in the control group. However, in the group with EAE treated with IFNβ-1b + melatonin, the TNFα and IL-1β levels were statistically similar to those observed in the control group.

On the other hand, in Figure 3C, it can be observed that the plasma IL-6 levels in the EAE group were significantly higher than in the nonimmunized control group. However, such an increment was significantly abolished by GA and IFNβ-1b treatments, and completely abolished by the treatments containing melatonin.

### 2.3. Oxidative Stress Markers

**Figure 4:**
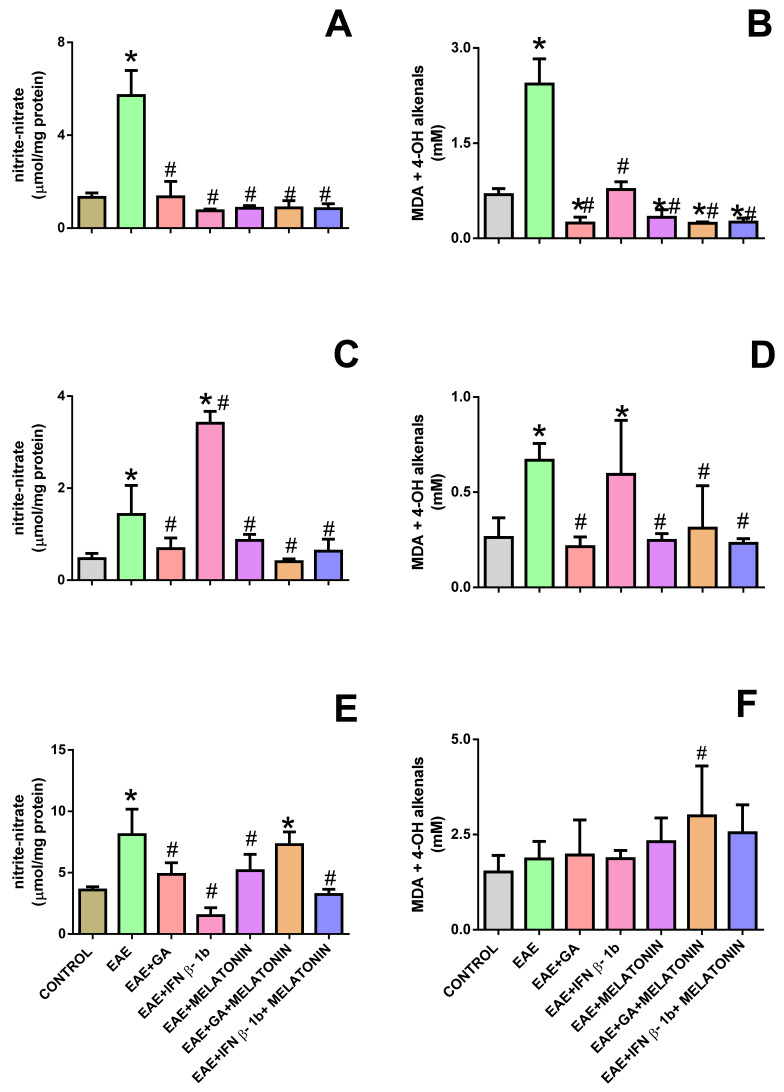
Levels of nitrite—nitrate (**A**–**C**) and end-products of lipid peroxidation (**D**–**F**) in the motor cortex (**A**,**D**), midbrain (**B**,**E**), and medulla homogenates from rats subjected to the indicated treatments. * indicates a significant difference from the control group (*p* < 0.05); # indicates a significant difference from the EAE group (*p* < 0.05).

In Figure 4, the respective levels of nitrite + nitrate, as well as the end-products of lipid peroxidation, are shown; Figure 3A,B show the respective levels in the motor cortex, Figure 4C,D present such levels in the midbrain, and Figure 4E,F presents the values in the spinal cord.

In the motor cortex, the EAE induction produced a significant increment in both parameters (nitrite–nitrate and end-products of lipid peroxidation), which was completely eliminated by all tested treatments. As in the motor cortex, the EAE induction produced a statistically significant increment in both parameters in the midbrain, which was eliminated by most treatments (except for the IFNβ-1b treatment).

In the spinal cord, as in the previous samples, the nitrite–nitrate level was significantly increased in the EAE group (Figure 4C), and such an increment was prevented in most tested therapeutic treatments (except for the melatonin–GA treatment). Otherwise, the level of end-products of lipid peroxidation were similar among the different tested groups (Figure 4F).

**Figure 5:**
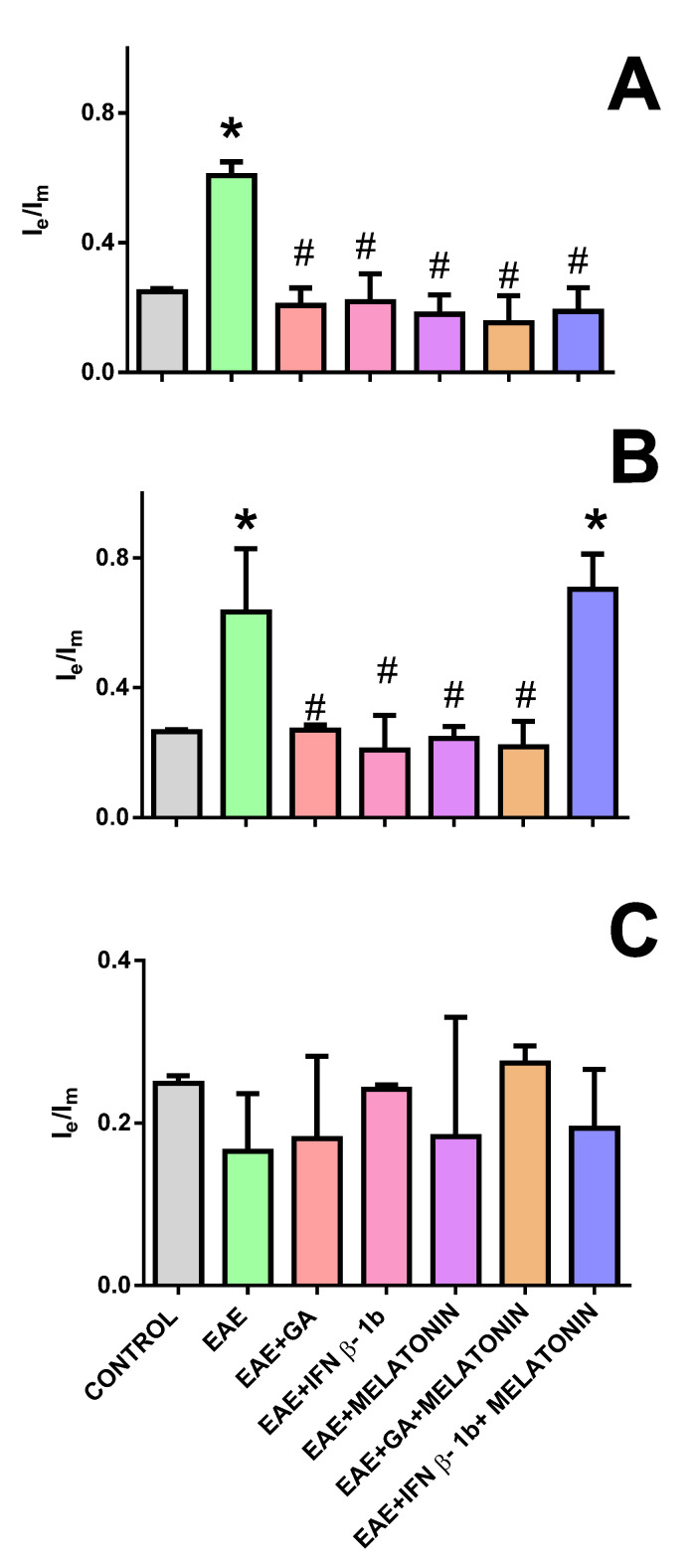
Membrane fluidity in motor cortex (**A**), midbrain (**B**), and spinal cord (**C**) homogenates from rats subjected to the indicated treatments. * indicates a significant difference from the control group (*p* < 0.05); # indicates a significant difference from the EAE group (*p* < 0.05).

When analyzing the fluidity of cell membranes by considering the Ie/Im ratio, it can be observed that, in the motor cortex and midbrain of EAE rats, there was a hyperfluidification of cell membranes (Figure 5A,B). In the motor cortex, all therapeutic treatments prevented this change in membrane fluidity (Figure 5A). However, in the midbrain, the melatonin - IFNβ-1b treatment was unable to prevent such an increment (Figure 5B). Lastly, in the spinal cord, the EAE induction, as well as the tested treatments, did not produce significant changes in the fluidity of membrane cells (Figure 5C).

## 3. Discussion

Our data showed that the crude CNS tissue homogenate used elicited a monophasic remitting/non-relapsing course of EAE. Similar results were obtained using SJL male mice and Lewis rats immunized with proteolipid 139-51 [28] and myelin basic protein, respectively [29]. Other subtypes such as chronic non-remitting EAE [30,31], benign EAE, and relapsing/remitting EAE were previously described [32,33]. These data show that the disease course of EAE is dependent on the strain, sex, and induction protocol.

Demyelination and inflammation induced by the entry of lymphocytes and macrophages into the CNS during the course of EAE have been reported [34]. In fact, infiltration of leukocytes into the CNS and the subsequent activation of resident glial cells and concomitant production of wide varieties of inflammatory molecules are neuroinflammatory key events in EAE. The ongoing inflammation is manifested by clinical signs, such as paresis and paralysis of the limbs.

Results obtained in our research showed that melatonin diminished the increase in proinflammatory cytokines in serum (IL1β, IL6, and TNFα) elicited by EAE. Our data are in line with previous reports in which melatonin was able to decrease the number of Th17 cells in the spleen, lymph nodes, and CNS; it also slowed the formation of pathologic cytokines associated with these cells [35]. In addition, melatonin decreases IFN-γ expression in the spinal cord and enhances splenic IL-10 expression in regulatory T cells by inducing IL-27 expression in the splenic dendritic cells; it also suppresses the expression of IFN-γ, IL-17, IL-6, and chemokine ligand 20 in the CNS and inhibits antigen-specific T-cell proliferation [36]. Melatonin also acts by altering the T effector/regulatory balance, which is associated with a reduced mononuclear infiltration (CD4 and CD11b cells) [24]. Furthermore, melatonin causes nonspecific immunity by inducing the production of natural killer cells [37] and IL-4 [38], which are important mediators in the amelioration or prevention of EAE-induced paralysis [4], as well as induces the repression of ICAM-1 expression in the spinal cord. In this regard, suppression of cell adhesion molecules is an important factor in reducing cell infiltration into CNS tissues [39,40]. Adhesion molecules are important in the trafficking of peripheral leukocytes into the CNS, which is a major event in the pathogenesis of MS [41]. Furthermore, melatonin might also reduce the activity of matrix metalloproteinase-9 and metalloproteinase-2, which have been implicated in disruption of the blood–brain barrier in MS. Interestingly, the decrement in metalloproteinase-9 and metalloproteinase-2 activities was associated with a reduced expression of TNF-α [42].

The mechanisms via which melatonin produces a cytokine decrement may involve the inhibition of the expression of nuclear factor κb (NF-κB) [43]. Furthermore, modulation of NF-κB by means of melatonin decreases the expression of inducible nitric oxide synthase and cyclooxygenase 2. Furthermore, it limits the production of prostanoids and leukotrienes, as well as other mediators of the inflammatory process [44,45].

The effects of melatonin on diminishing the levels of the stable products of lipid oxidation and on nitric oxide release in the motor cortex, midbrain, and spinal cord were similar to those induced by GA and IFNβ-1b. Additionally, our data showed that melatonin, GA, and IFNβ-1b preserved the fluidity of cell membranes in the indicated brain areas. Therefore, the possible changes produced by oxidative stress and inflammation in the conformation and activity of membrane-bound enzymes and signal transduction pathways are avoided.

Melatonin, GA, and IFNβ-1b have different modes of action. The effect of GA involves multiple cell types, including antigen-presenting cells, being proposed to modulate the immune system in many ways, such as by diminishing IL-17 and IFN-γ synthesis by peripheral blood mononuclear cells and CD4^+^ T cells [46]. GA therapy during the chronic phase of EAE leads to increased proliferation, differentiation, and survival of oligodendrocyte precursor cells [47]. IFNβ regulates immune cell activation and proliferation, autoreactive T-cell apoptosis, induction of anti-inflammatory cytokine shifts, inhibition of immune cell trafficking across the blood–brain barrier, and antiviral activity [48].

The side-effects of IFNβ-1b include flu-like symptoms, menstrual disorders in women, a decrease in neutrophil count and white blood cell count, an increase in amino transferase levels, and development of neutralizing antibodies to IFN-β [49]. Flushing, chest pain, palpitations, urticaria, and dyspnea are possible side-effects related to injection of GA, but the latter might also occur without any temporal relation. In addition, recent evidence has suggested the possibility of hepatoxicity [50]. In contrast, melatonin has no known toxic dose level in humans or animals; a dose of 800 mg/kg did not cause death in mice [51]. Moreover, the capability of melatonin to repair the neuronal damage caused by free radicals together with its anti-inflammatory properties confirm that melatonin has several advantages over IFNβ-1b and GA.

Our results show that melatonin alone or in combination with GA and IFNβ-1b administration delays the onset of paralysis and diminishes the clinical score, together with decreases in the synthesis of proinflammatory cytokines and oxidative stress markers in a rat model of EAE.

Our data on the effects of melatonin on proinflammatory cytokines and oxidative stress markers are in accordance with the results recently reported in a model of relapsing/remitting EAE [33]. In addition, our results showed that EAE causes a hyperfluidification of cell membranes in the motor cortex and midbrain. This change may have clinical relevance, since it has been suggested that alterations in the physicochemical properties of cell membranes are linked to stress responses [52] and could influence cytosolic reactive oxygen levels and mitochondrial activity [53]. Thus, the effects of melatonin and other therapeutic treatments on cell membrane fluidity in the motor cortex and midbrain warrant further investigation.

## 4. Materials and Methods

### 4.1. Animals

Male adult Sprague-Dawley rats, weighing 220–250 g, obtained from the animal facility at the Western Biomedical Research Center, Mexican Institute for Social Security (Guadalajara, México), were kept inside plastic cages maintained in a temperature- and humidity-controlled environment, wherein 12 h light–dark cycles were implemented. Animals were fed with standard rat food, and they had free access to tap water.

### 4.2. EAE Induction, Treatments, and Clinical Score Assessment

Rats were immunized by a single intraperitoneal injection of a homogenate of spinal cord and pig brain (25 mg each one) suspended in sterile physiological saline solution and emulsified with 50 μL of complete Freund’s adjuvant acquired from Sigma Chemical Co. (St. Louis, MO, USA) containing 1 mg/mL heat-killed *Mycobacterium tuberculosis* strain H37Ra.

Seventy-two immunized animals (EAE) were randomly assigned to six experimental groups (*n* = 12 per group) as shown below.

Group 1 (control group with EAE): EAE rats, treated only with an i.p. injection of the vehicle.

Group 2 (EAE—GA): EAE rats, treated with GA (10 mg/kg of body weight/day); the first GA i.p. injection was performed at 8:00 a.m., 1 h after the EAE induction.

Group 3 (EAE—IFNβ-1b): EAE rats, treated with IFNβ-1b (9000 IU/kg of body weight every other day); the first IFNβ-1b i.p. injection was performed at 8:00 a.m., 1 h after the EAE induction.

Group 4 (EAE—melatonin): EAE rats, treated with melatonin (20 mg/kg of body weight/day); the first melatonin i.p. injection was performed at 8:00 a.m., 1 h after the EAE induction.

Group 5 (EAE—melatonin + GA): EAE rats, treated with melatonin (20 mg/kg of body weight/day; the first melatonin i.p. injection was performed at 8:00 a.m., 1 h after the EAE induction), 1 h after a GA i.p. injection was administrated (10 mg/kg of body weight/day).

Group 6 (EAE—melatonin + IFNβ-1b): EAE rats, treated with melatonin (20 mg/kg of body weight/day; the first melatonin i.p. injection was performed at 8:00 a.m., 1 h after the EAE induction), 1 h after a IFNβ-1b i.p. injection was administrated (9000 IU/kg of body weight every other day).

A control group of non-EAE-immunized rats (*n* = 12) received daily injections of vehicle (control).

A stock solution of melatonin (5% *w*/*v*) was dissolved in a multicomponent solvent containing ethanol, propylene glycol, monopropylene glycol, and water (10/25/20/45 *w*/*w*/*w*/*w*).

Rats were observed daily for assessment of the severity and extent of motor function deficits according to the following score: 0, healthy; 1, floppy tail; 2, mild paraparesis; 3, severe paraparesis; 4, tetraparesis; 5, moribund or dead. On day 10 post immunization (at the peak of clinical signs), eight rats from the control and experimental groups were euthanized for biochemical analysis. The remaining rats were monitored every day until 20 days post immunization. Severity of disease as a cumulative disease index score was calculated by adding the daily disease activity scores over the 20 days of the experiment.

### 4.3. Biochemical Analysis

Four rats from each experimental group were used for the quantification of the oxidative stress markers and plasma proinflammatory cytokines. Rats were euthanized by decapitation, and their brains were rapidly removed from the skull. Tissues were dissected on humidified filters at 4 °C and were rapidly homogenized with ice-cold phosphate-buffered solution (PBS), containing a complete protease inhibitor cocktail (Sigma-Aldrich) and ethylenediaminetetraacetic acid (EDTA; 5 mM), using a Potter–Elvehjem tissue grinder to produce 1:10 (*w*/*v*) homogenates. Homogenates were centrifuged at 3000× *g* for 15 min at 4 °C, and the supernatant was collected and stored at −80 °C until assayed.

Peripheral venous blood was collected into Vacutainer K2-EDTA tubes (BD). Blood was centrifuged at 3500 rpm for 5 min to separate the plasma. Plasma was stored at −80 °C until analysis. Protein concentration was determined using Lowry’s method with bovine serum albumin as the standard [54].

Plasma levels of TNFα, IL-1β, and IL-6 were measured by a sandwich-type enzyme-linked immunosorbent assay (ELISA) technique, using Kits from R&D Systems (TNFα DTA00C (detection range: 15.6–1000 pg/mL), IL-1β DLB50 (detection range: 3.9–250 pg/mL), and IL-6 D6050 (detection range: 3.12–300 pg/mL)), according to the manufacturer’s instructions.

Nitrite–nitrate values were quantified as an index of nitric oxide production. Briefly, the sample was deproteinized with zinc sulfate, as reported elsewhere [55], followed by centrifugation at 14,000× *g* for 15 min. Then, 100 µL of the supernatant was applied to a microplate well, followed by the addition of 100 µL of vanadium(III) chloride (0.8%) to each well in order to convert nitrate to nitrite. Subsequently, the Griess reagents were added (50 µL of sulfanilamide (2%) and 50 µL of *N*-(1-naphthyl) ethylendiamine dihydrochloride (0.2%)). After 30 min of incubation at 37 °C, absorbance was read at 540 nm [56]. The nitrite concentration of the samples was calculated by plotting a standard NaNO_2_ curve (1–100 μM).

The level of lipid peroxidation end-products (LPO) was measured as the sum of malondialdehyde (MDA) plus 4-hydroxyalkenals (4-OHA) via a colorimetric method, using an LPO Cuvette Based Assay Kit from Oxford Biomedical Research, Inc. (Oxford, MI, USA); each measurement was repeated at least three times, according to the manufacturer’s instructions.

Membrane fluidity was estimated from the excimer-to-monomer fluorescence intensity ratio (I_e_/I_m_) of the fluorescent probe 1,3 dipyrenylpropane (DPyP) incorporated into membranes, as reported elsewhere [57].

### 4.4. Statistical Analysis

Results are expressed as the mean ± SD. One-way ANOVA followed by Bonferroni’s post hoc comparison was performed in all statistical analyses. Statistics with a value of *p* < 0.05 were considered significant.

## 5. Conclusions

Administration of melatonin ameliorated immune-mediated CNS damage, delaying its onset and progression, reducing the maximal disease score and the cumulative index score, and decreasing inflammation and oxidative stress markers in a rat model of MS. The effects of melatonin on oxidative stress markers and proinflammatory cytokines were comparable to those of GA and IFNβ-1b. The proposed protective mechanisms are possibly related in each case to their respective anti-inflammatory and/or antioxidant properties.

Our data demonstrate the protective role of melatonin in EAE; in particular, we found antioxidant and anti-inflammatory effects. This data may be useful to find new strategies with possible treatment applications for neurodegenerative diseases. For example, the effect of dietary consumption of fruits and seeds that contain significant levels of melatonin and other bioactive compounds can be investigated. In this regard, it is well known that dietary melatonin, particularly from plant sources, leads to a significant increase in its serum levels, and this may have a beneficial impact on the central nervous system, as melatonin has the ability to cross the blood–brain barrier. Additionally, melatonin has an uncommonly high safety profile, which may be advantageous compared to other natural compounds.

## Data Availability

The data presented in this study are available upon request from the corresponding author.

## Sample Availability

Samples of the compounds are not available from the authors.
